# Toward process-based inventory data for Portland clinker substitutes

**DOI:** 10.1007/s44498-026-00186-x

**Published:** 2026-09-18

**Authors:** Pippa Edwards, Wilson Ricardo Leal da Silva, Paul Fennell, Rupert J. Myers

**Affiliations:** 1 Department of Civil and Environmental Engineering, Imperial College London, London, SW7 2AZ UK; 2 Fuller Technologies, Copenhagen, Denmark; 3 Department of Chemical Engineering, Imperial College London, London, SW7 2AZ UK

**Keywords:** Inventory data, Life cycle assessment, Clinker substitutes, Supplementary cementitious materials, Low carbon cement

## Abstract

The cement industry must produce lower carbon cements to meet its 2050 climate goals. A key low-cost strategy to achieve this is to substitute Portland clinker in cement with lower carbon materials. However, high-quality inventory data for clinker substitutes are poorly available, which limits the application of life cycle assessment (LCA) to quantify the carbon footprints of cements containing them. Here, we report the state-of-the-art inventory data for clinker substitutes, disaggregating them by unit process to facilitate comparison, improve proxy data selection, and identify gaps to inform future data collection efforts. Cradle-to-gate greenhouse gas emissions for European production of the clinker substitutes and carbon footprints of composite cements defined in EN 197 are calculated. Overall, we find the degree of clinker substitution, rather than the processing of clinker substitutes, is more impactful for producing low carbon footprint cements. This work can improve the transparency of LCA studies of concrete materials, verify commercial data, and inform technology development and business decisions related to the concrete value chain.

**Supplementary Information:**

The online version contains supplementary material available at https://doi.org/10.1007/s44498-026-00186-x.

## Introduction

Portland cement products are essential building materials but their production is responsible for 7–8% of anthropogenic CO_2_ emissions (Miller et al., 2018). This is primarily due to Portland clinker production which releases CO_2_ from limestone calcination and fuel combustion at high temperatures (~ 1450 °C) (Habert et al., 2020; Taylor, 1997). Portland clinker substitution is a key strategy for cement industry decarbonisation. For example, increased utilisation of clinker substitutes to produce composite cements can reduce cement CO_2_ emissions by up to 1.3 Gt per year (Shah et al., 2022) due to their lower carbon footprints. Many clinker substitutes are already industrially proven and comprise a great share of commercial cement products in the market, with conventional materials such as blast furnace slag, coal fly ash, and limestone currently displacing ~25 wt.% of clinker in Portland cements on average globally (GCCA, 2024).

Clinker substitution accounts for 9% of the cement and concrete emissions reduction pathway in the GCCA (Global Cement and Concrete Association) 2050 net zero roadmap, which targets average clinker contents in cement of 58 and 52 wt.% by 2030 and 2050 respectively (GCCA, 2021). However, CO_2_ emissions reduction from clinker substitution is threatened by the potentially declining availability of coal fly ash and blast furnace slag that may be caused by decarbonisation of the electricity, and iron and steel sectors (Habert et al., 2020). Thus, commercialisation of other materials that can be produced at scale is important to realise emissions reduction from clinker substitution, with emerging materials such as calcined clays, recycled concrete fines, and biomass ashes attracting significant research attention (e.g. (F. H. Avet, 2017; Carriço et al., 2021; Chaunsali et al., 2018; Oksri-Nelfia et al., 2016; Scrivener et al., 2018a, 2018b)).

Increasing the supplies of viable clinker substitutes will require increased levels of processing (Snellings et al., 2023); whether during extraction (e.g., quarrying, crushing), reactivity beneficiation (e.g., calcination, comminution), purification (e.g., washing or leaching processes), or preparation for packaging/transport (e.g., drying, classification) (Snellings et al., 2023). However, the environmental impacts of processing clinker substitutes are not consistently considered in technology roadmaps (e.g. GCCA roadmap (GCCA, 2021)), life cycle assessment (LCA) studies (Shah et al., 2022), and the IPCC methodology for estimating CO_2_ emissions from cement production (IPCC, 2000). Environmental impacts from clinker substitute processing have been excluded from assessment of composite cements for multiple reasons, including where studies have assumed these impacts make minor contributions to current cement production CO_2_-eq. emissions (Damineli et al., 2010; Habert & Roussel, 2009; Shah et al., 2022). Other studies include the impacts of clinker substitutes using aggregated emission factors such as global warming potential (e.g., kg CO_2_-eq. tonne^−1^ cement (Flower & Sanjayan, 2007)), or energy consumption (e.g., MJ tonne^−1^ (Gettu et al., 2019)), rather than in the higher level of detail that critical process feedstocks are normally considered in process-based LCA. Excluding clinker substitute processing can overestimate the emissions reduction potential composite cements, obscuring understanding of their genuine environmental performance, and overlooks the contributions of elementary flows in non-climate change impact categories (e.g., PM (particulate matter) emissions).

LCA is used to estimate the environmental impacts of products and services across their life cycles (including raw material acquisition, production, use, and end-of-life including recycling and final disposal) (British Standards Institution, 2020). LCA involves four phases; (1) goal and scope definition; (2) inventory analysis; (3) impact assessment; and (d) interpretation. The inventory analysis phase quantifies the inputs and outputs of the product or service and is often iterated as more information is gathered about the system (British Standards Institution, 2020). Inventory analysis can be time consuming, particularly when collecting primary inventory data (British Standards Institution, 2020). Different approaches have been proposed to reduce the data collection burden, or to assist where primary data do not readily exist (e.g., industrial scale data for emerging technologies). In such cases, inventory data can be approximated by proxy from similar systems (‘proxy technology’) or constructed leveraging domain knowledge (e.g., process modelling) (Georgiades et al., 2025; Tsoy et al., 2020).

Representative inventory data is essential for generating reliable and correct insights from LCA studies, e.g., identifying low carbon materials and processes. Uncertainty of inventory data can be model-related (fluctuations in process stability, measurement error) or contextual (e.g., difference in regions, technology type, or age) (Zargar et al., 2022). Hung and Ma (2009) used Monte-Carlo analysis to determine that inventory data had the highest contribution to overall LCA result uncertainty for a case study of municipal solid waste management in Taiwan. They also found that that the uncertainty varies between impact categories (Hung & Ma, 2009). Weidema and Wesnaes (1996) proposed a ‘pedigree matrix’ methodology to semi-quantify the uncertainty of inventory data considering their ‘pedigree’: reliability, completeness, and temporal, geographical, and technological representativeness (Weidema & Wesnæs, 1996).

There are at least three notable publicly accessible sources of inventory data for clinker substitutes: the GreenConcrete webtool (Gursel, 2012), the OpenConcrete tool (Kim et al., 2022), and The Inventory of Carbon and Energy (ICE) Database (Circular Ecology, 2024). The GreenConcrete webtool includes inventory data for coal fly ash, granulated blast furnace slag, natural pozzolans, and limestone, but does not include data for other materials approved per the EN 197 (British Standards Institution, 2019) series such as calcined clay and recycled concrete fines (RCF). The OpenConcrete tool includes inventory data for the same materials as GreenConcrete with the addition of calcined clays. The ICE Database, which is intended for assessing the whole life-cycle of buildings, provides data for the same four clinker substitutes as GreenConcrete (coal fly ash, granulated blast furnace slag, natural pozzolans, and limestone), but only provides a pre-aggregated emissions factor for each material (e.g., kg CO_2_-eq. kg^—1^ material) instead of a process-based inventory detailing inputs and outputs. There is also commercially-available data, such as that included in databases such as ecoinvent (Kellenberger et al., 2007), and the GCCA EPD (Environmental Product Declaration) tool (GCCA, 2026), which includes pre-calculated aggregated emissions factors for clinker substitutes including GBFS, fly ash, silica fume, calcined clay, limestone, pozzolans, and RCF. Other works investigating the processing contributions of clinker substitutes have used inventory data from a variety of sources, including site surveys (e.g., (Marceau & VanGeem, 2003; Rosado et al., 2017)), estimates from engineering studies and manufacturer datasheets (e.g., Chen et al., 2010; Habert et al., 2011; Heath et al., 2014; Meryman, 2009)), and experimental or modelling data (Salvi Malacarne et al., 2021; Tasiopoulou et al., 2023; Vargas et al., 2020). Additionally, life cycle data for specific cement and concrete products are increasingly published by commercial producers in the form of EPDs, and include the production, in use, and disposal/recycling life cycle stages. However, the data reported in EPDs are pre-aggregated as specific impact category indicators (e.g., GWP), and do provide detailed information about the underlying process information to support detailed life cycle assessments (e.g., contribution analysis). Therefore, there is a gap in flexible and comprehensive inventory data available to support the investigation of a range of common and emerging clinker substitutes using LCA.

To address such gap, this paper aims to provide an accessible and more complete set of inventory data for clinker substitutes than that which currently exists, notably for the improvement of future LCA studies. We assess the quality of the available data, identify gaps or improvements in the available data to guide future data collection, and disaggregate the data into unit process datasets to improve their utility. We conduct LCA using the collected inventory data to identify the processes and products with the lowest carbon footprint and examine the implications for producing low carbon cements.

## Methodology

### Database scope

Inventory data were collected from the literature for selected clinker substitutes and disaggregated by unit processes (e.g., drying, calcination, milling). The data were converted into consistent units to allow comparability, and their quality was assessed using the data pedigree.

The materials considered in scope included granulated blast furnace slag (GBFS), coal fly ash, steel slag, natural pozzolana, silica fume, calcined clays, limestone, bauxite residue, biomass ash, mine tailings, and RCF (where both uncarbonated RCF and carbonated RCF, denoted cRCF, are considered). These materials are either established clinker substitutes approved for use in composite cements per EN 197 (British Standards Institution, 2019), or materials under frequent investigation in the literature for use as clinker substitutes ((Bernal et al., 2016; Juenger et al., 2019; Lothenbach et al., 2011; Scrivener et al., 2018a; Shah et al., 2022; Snellings et al., 2023)).

### Data collection

**Fig. 1:**
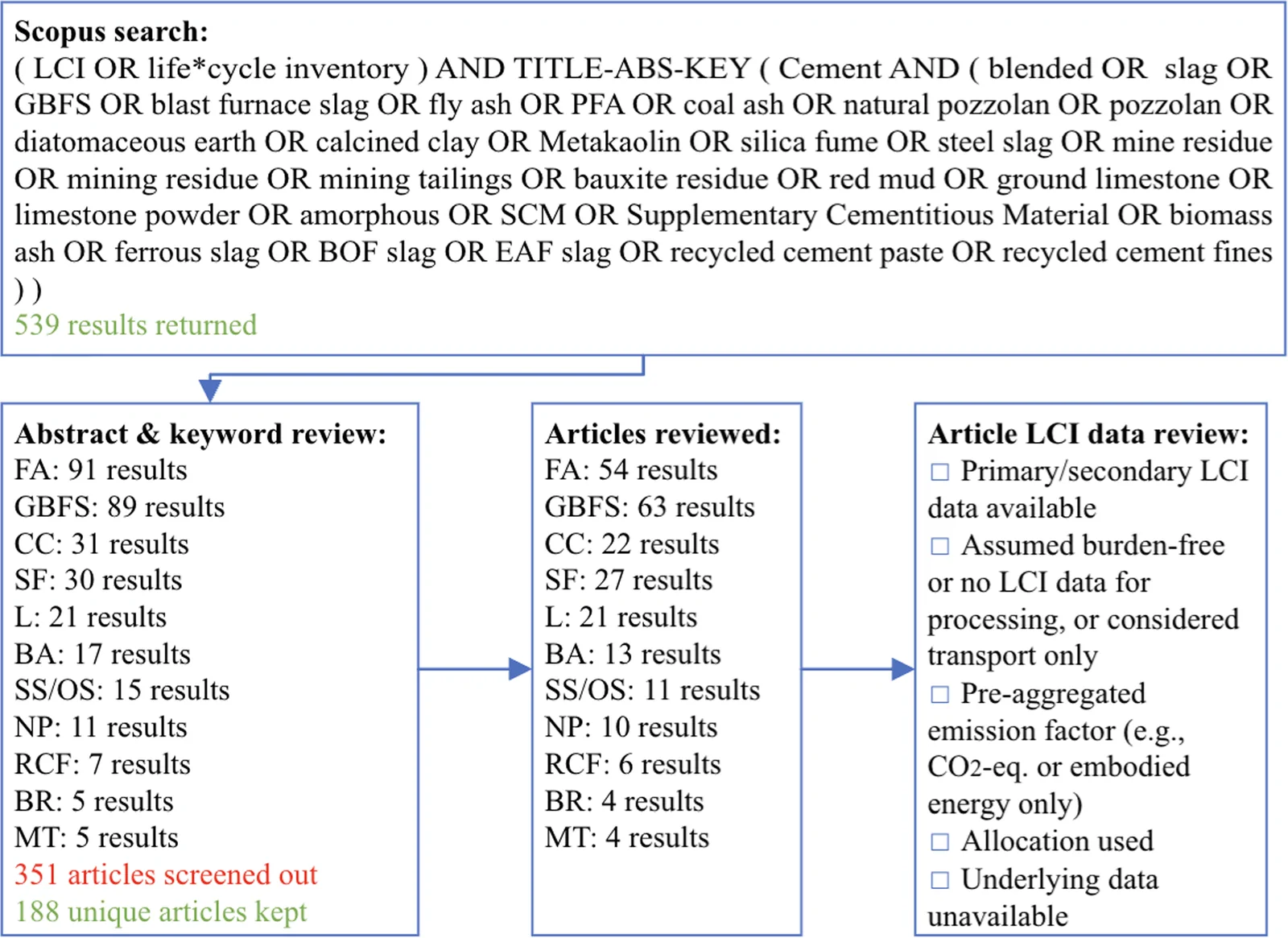
Methodology for Scopus literature search. FA fly ash, GBFS granulated blast furnace slag, CC calcined clay, SF silica fume, L limestone, SS/OS steel slag/other slags, NP natural pozzolans, RCF recycled cement fines, BR bauxite residue, MT mine tailings

Process-based inventory data, which describes the input and output of materials and energy for a given process system, were collected from a review of the scientific literature using Scopus and the search term as per Fig. 1. Other inventory data known to the authors were added to supplement the results from Scopus. Where secondary data were used in a study, their original source(s) were identified, and data were obtained directly from them if available.

The input and output of materials and energy included intermediate flows (e.g., material and energy transferred between activities such as electricity, heat energy, coking coal) and elementary flows (e.g., material and energy transferring between activities and the environment, e.g., CO_2_, water vapour, PM emissions). References 'et al., 2018, Environment Agency, 2023' are cited in text but not provided in the reference list. Please provide references in the list or delete these citations. All processing stages (e.g., drying, calcining, milling) between the points of generation and/or collection for each material, to their points of use in cement, were included and represented the ‘foreground system’ for a given clinker substitute. All supporting processes related to intermediate flows used by the foreground system (e.g., electricity production) were considered part of the ‘background system’.

### Data transformation

We disaggregated the collected inventory data into unit process datasets from the study scope and system descriptions. Data for single processing stages, e.g. crushing, were designated as such. Where disaggregated data described multiple processing stages, a ‘main’ process was designated with all other processes considered ‘minor’ processes. For example, for data describing the crushing and washing of a material; ‘crushing’ was designated the main process, and ‘washing’ the minor process.

All intermediate and elementary flows were converted into SI units. Fuel consumption was converted to MJ of heat supplied (see Supplementary Information SI2 for the conversion factors used), and elementary flows associated with combustion were removed from the foreground system to prevent double counting with elementary flows associated with the background heat flows. The disaggregated process data per 1 tonne reference product are available as block flow diagrams, with both economic and elementary flows represented, in Supplementary Information SI2.

### Quality assessment

We used the pedigree matrix approach (Weidema & Wesnæs, 1996) (refer Supplementary Information SI1, Section S1, Table S1) to semi-quantitatively assess the collected inventory data. Scores for each pedigree matrix category (reliability, completeness, temporal correlation, geographical correlation, and further technology correlation) were applied from 1–5, with 1 being the strongest (e.g., most reliable) and 5 being the weakest (e.g., least reliable). Geographic and technological scores were relative to the scope of this study, e.g., European production of materials for use as clinker substitutes in cement. Descriptions of the inventory data, detailing the data year, source of data (e.g., measured from operational site, calculated from process model), number of sites, region, and technological relevance to clinker substitute processing, and the pedigree scores, are provided in Supplementary Information SI2, Section S1.

### Life cycle assessment

#### Goal and scope

This study evaluates the carbon footprint of processing clinker substitutes for use in composite cement. We considered both a gate-to-gate scope (Sect. 4.1.1) for the frequently recorded unit processes (> 3 times), and a cradle-to-gate scope for the highest scoring inventory data for each material. We consider generic European production, to align with the EN 197 (British Standards Institution, 2019) composite cement scope. The IPCC 2021 Global Warming Potential (GWP100) impact assessment method was used to determine carbon footprint of each material, with all calculations performed in Activity Browser version 2.11.2 (Steubing et al., 2020). Finally, the LCA results were compared to performance as clinker substitute (i.e., reactivity determined using the R^3^ test per Sect. 2.6), using both reactivity data and approved composite cement mix designs per EN 197.

##### Gate-to-gate (unit process) assessment

A gate-to-gate LCA was conducted to compare the frequently occurring (e.g., recorded >3 times in the collected data) unit processes for producing clinker substitutes: calcination, drying (heat-fired), milling, crushing, and quarrying (extraction). A functional unit of 1 tonne of product was used. We used coal as the fuel source for process heat (e.g., for calcination and drying processes, excluding diesel-derived heat), based on heat-source having the largest contribution to results (Supplementary Information SI1, Section S5), to compare processes and datasets using a consistent single fuel basis.

##### Cradle-to-gate (materials for use as clinker substitute) assessment

A cradle-to-gate LCA was conducted to compare the clinker substitutes for use in cement, with upstream processing impacts excluded for by-product derived clinker substitutes per the applied cut-off approach. A functional unit of 1 tonne clinker substitute processed for use in cement was used. For each material, we considered the inventory data with the highest scoring data pedigree (per Sect. 2.4, summarised in Table 1), prioritising the technological pedigree score (which represents whether the technology processes the material for use as a clinker substitute in cement) and the reliability pedigree score. We compare three different sources of process heat: coal, natural gas and refuse derived fuel (RDF) (see Sect. 4.3 Inventory analysis), as process heat from coal was found to have the highest contribution to carbon footprint (refer to Supplementary Information SI1, Section S5).

**Table 1:** Foreground unit process datasets used for cradle-to-gate

Material	Dataset in supplementary information SI2	Processes included
Fly ash	1.1	Drying
GBFS	2.1	Granulation, drying, crushing, milling
Calcined clay	3.5	Quarry extraction, calcination, milling
Limestone	4.1a	Quarry extraction, crushing milling
Natural pozzolan	5.3	Quarry extraction
RCF, cRCF	6.4, 6.5	6.4: Crushing, separation (RCF, cRCF) 6.5: Semi-dry carbonation (cRCF only)
Biomass ash	7.1	Milling
Steel slag	8.3	Screening, crushing, sieving Median result for milling from Sect. 4.1.1 results added due to lack of steel slag milling unit process dataset
Bauxite residue	9.1, 9.2	9.1: Dewatering (filter press) 9.2: Vitrification
Mine tailings	10.1a/b	Drying, milling, calcination Average of 10.1a/b taken

LCADataset reference numbers in the second column refers to the numerical reference for each unit process dataset per Supplementary Information SI2

#### Inventory analysis

The collected unit process datasets were used as foreground data (refer to Sect. 2.5.1.1, 2.5.1.2). Background data (Table 2) were taken from ecoinvent v3.9.1 (Swiss Centre for Life Cycle Inventories, 2022) unless otherwise specified. We used inventory data for RDF fired heat based on IPCC data which assumes that the calorific value of municipal waste is derived from 60% biomass and 40% non-biomass (Eggleston et al., 2006), and upstream RDF processing (e.g., shredding) in based on the inventory from (Georgiades et al., 2023). We note that RDF is most likely used as a partial (e.g., 40% replacement of coal) rather than complete fuel source; however, we consider 100% RDF to demonstrate the possible lower bound. Economic allocation was performed for multi-functional systems (i.e., where processes had more than one product e.g., RCF production, refer to Supplementary Information SI1, Section S2.2 for detailed discussion). The cut-off approach was used to exclude emissions from upstream processes (e.g., pig iron production, coal-fired electricity production). Cut-off is generally only permitted when the contribution is negligible, which is not necessarily the case for pig-iron production and coal-fired electricity production. However, the upstream processes impacts and allocation contribution have been previously reported by Chen et al. (2010), using fixed prices assumptions. As our scope is on the processing of material for use as clinker substitutes, we have avoided embedding fixed price assumptions where possible in this result by excluding the impact of upstream processes. We note that LCA practitioners incorporate these upstream impacts using the existing published data by Chen et al. (2010) and relevant price data at time of study.

**Table 2:** Background data used in the LCA calculations, both gate-to-gate and cradle-to-gate scopes

Flow	Description	Region	Source
Electricity	Market group for electricity, medium voltage	Europe without Switzerland	Ecoinvent 3.9.1
Water	Market for tap water	Europe without Switzerland	Ecoinvent 3.9.1
Wastewater treatment	Treatment of wastewater from ground granulated blast furnace slag production, wastewater treatment	RoW	Ecoinvent 3.9.1
Residue from mechanical treatment	Market for residue from mechanical treatment, industrial device	RoW	Ecoinvent 3.9.1
Blasting / explosives	Blasting	RER	Ecoinvent 3.9.1
Lubricating oil	Lubricating oil production	RER	Ecoinvent 3.9.1
Petroleum coke	Petroleum coke production	Europe without Switzerland	Ecoinvent 3.9.1
Silica sand	Market for silica sand	GLO	Ecoinvent 3.9.1
Bentonite	Market for bentonite	GLO	Ecoinvent 3.9.1
Diesel	Market for diesel, burned in building machine	–	Ecoinvent 3.9.1
Coal-fired heat	Heat production, at hard coal industrial furnace 1-10 MW	Europe without Switzerland	Ecoinvent 3.9.1
Gas-fired heat	Heat production, natural gas, at industrial furnace low-NOx >100 kW	Europe without Switzerland	Ecoinvent 3.9.1
RDF-fired heat	Determined from IPCC data from municipal waste containing 60% biomass and 40% non-biomass (Eggleston et al., 2006)	N/A	(Eggleston et al., 2006), (Georgiades et al., 2023)

#### Composite cement emissions

We assessed the carbon footprint (kg CO_2_-eq. tonne^—1^) of composite cements made with the approved clinker substitutes (e.g., limestone, natural pozzolans, fly ash, GBFS, calcined clay and RCF) per EN 197 (British Standards Institution, 2019), and additionally include LC3-20 (limestone calcined clay with clinker factor of 20%) as a low-clinker composite cement of interest. The composite cements are assessed on a mass basis which does not imply functional equivalence (e.g., same performance in concrete). Composite cement CO_2_-eq. emissions were calculated using Eq. (1), which is based on 100% cement mix design per Table 1 of BS 197–1 (i.e., which excludes gypsum, that is added up to 5wt.% in addition to 100% shown in the table for the purpose of setting control).

1 $$ {{GWP}}_{{{{cement}}}} = {{f}}_{{{{clinker}}}} \cdot{{GWP}}_{{{{clinker}}}} + \left( {1 - {{f}}_{{{{clinker}}}} } \right)\cdot{{GWP}}_{{{clinker \, substitute}}} $$

where *f*_*clinker*_ is the clinker factor in cement (wt. % clinker in cement), *GWP*_*clinker*_ is the carbon footprint of clinker production (kg CO_2_-eq. tonne^—1^ clinker), *GWP*_*clinker substitute*_ is the carbon footprint of clinker substitute production (kg CO_2_-eq. tonne^—1^ clinker substitute). We excluded transportation of clinker substitutes as transport scenarios are variable depending on the specific production conditions and the scope of this work is to understand processing-based impacts rather than produce product-level LCAs, however we note that transport emissions should be included separately when applying the data from this study in cement-based LCA. We used the minimum clinker factor for each cement blend as *f*_*clinker*_, which resulted in 5 wt.% additional minor constituent as limestone being included for CEM I. A GWP_*clinker*_ value of 832.8 kg CO_2_-eq. was used for European production of clinker (refer to Supplementary Information SI1, Section S4). Global warming potentials for each approved clinker substitute were taken from the cradle-to-gate results for each material, using the coal and RDF-fired result as the maximum and minimum cases respectively. Where more than one clinker substitute was used (e.g., in LC3 cement), we calculated *GWP*_*clinker substitute*_ using Eq. (2):

2 $$ {GWP}_{clinker\, substitute} { = }\frac{\sum {m}_{clinker\, substitute, \, i} \cdot {GWP}_{clinker\, substitute, \, i}} {\sum {m}_{clinker\, substitute, \, i}} $$

where *m*_*i*_ is the mass of each clinker substitute, and *GWP*_*clinker substitute,i*_ the carbon footprint of each clinker substitute (kg CO_2_-eq. tonne^—1^ clinker substitute). We note that all GWP values used are the “gross” output of the cradle-to-gate result calculated using the IPCC 2021 GWP100 impact assessment method, with no additional adjustments applied to these results.

### Performance data for clinker substitutes

We collected 7-day heat release data (per the R^3^ heat release test (F. Avet et al., 2016)) for clinker substitutes from the literature (e.g., (Al-Shmaisani et al., 2022a; Frías et al., 2024; Giels et al., 2025; Hertel et al., 2022; Koufany et al., 2025; X. Li et al., 2018; Londono-Zuluaga et al., 2022; Ramanathan et al., 2021; Snellings, 2016; Sousa et al., 2024; Suraneni et al., 2019; Yao et al., 2024)) as a proxy to assess the potential performance of materials in cement. R^3^ heat release data was used due to the availability of results across different material types, although we note that this test is reported to favour materials with high alumina contents (e.g., (Al-Shmaisani et al., 2022b)). We used <160 J g^—1^ of heat released at 7-days as a measure of ‘low’ reactivity in cement, 160 – 350 J g^—1^ for ‘moderate’ reactivity in cement, and >350 J g^—1^ for ‘high’ reactivity in cement, based on the 90% confidence interval reported for pozzolans in (Londono-Zuluaga et al., 2022). Due to lack of published data for cRCF, we assumed the same reactivity as RCF based on preliminary analysis of carbonated RCF performance (Sreeram et al., 2025).

## Properties of the inventory data

### Overview

We reviewe 188 articles from the 539 results returned from the Scopus literature review (Fig. 1) to obtain inventory data for clinker substitutes. We identified 71 LCA studies that used pre-aggregated emission factors instead of process-based inventory data, which is less transparent and flexible. This is because primary contributions to the emission factor (e.g., fuel combustion) cannot be identified, nor can different scenarios (e.g., effect of decarbonising electricity grid) be easily tested.

We identified that the majority of LCA studies of GBFS (e.g., (Chen et al., 2010; Gursel, 2012; Kellenberger et al., 2007; Swiss Centre for Life Cycle Inventories, 2022)) used the same inventory data produced from a 2003 survey of U.S. producers (Marceau & VanGeem, 2003), highlighting the lack of unique inventory data sources for common clinker substitute materials. In total, 36 sets of inventory data were included, which were disaggregated into 57 unit process datasets. The number of collected inventory data for each material were: fly ash (1); GBFS (2); calcined clay (6); limestone (4); natural pozzolans (4); recycled concrete products (7); biomass ashes (4); steel slag (4); mine tailings (2); and bauxite residue (2). No processing data were collected for silica fume, as this product is typically not processed for use as a clinker substitute beyond collection, packaging, and transport.

### Process map and quality assessment

**Fig. 2:**
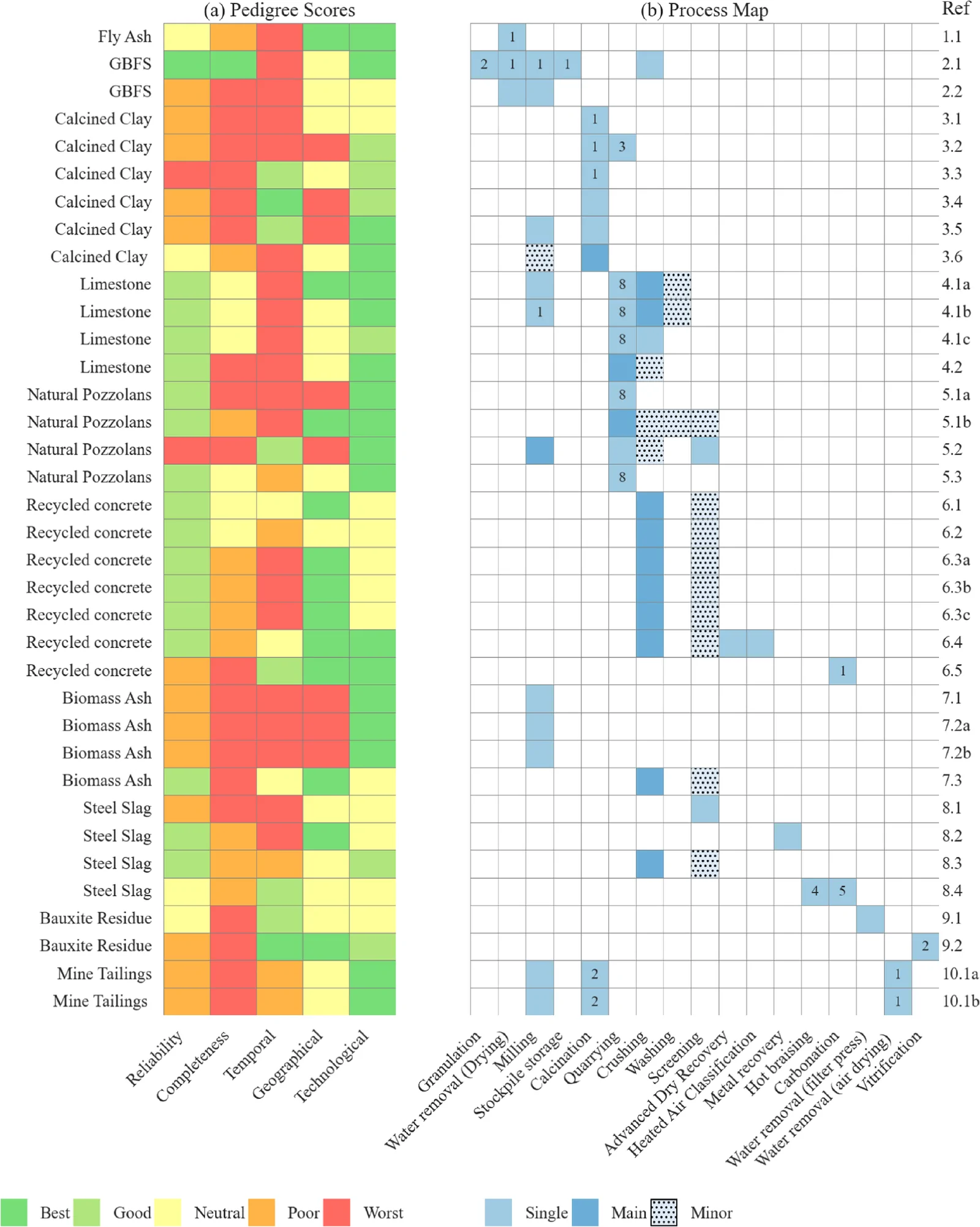
**a** Pedigree scores and **b** process mapping of the collected inventory data. Pedigree scores per each of the five categories (reliability, completeness, temporal correlation, geographical correlation, technological correlation) are represented by the colours green (best) to red (worst). Process designation is represented as: single processes (solid navy), main processes (solid blue), minor processes (dotted blue). Black numbers in (**b**) cells indicate the number of elementary flows specified in the foreground system of that process. Cells with no numbers indicate that no elementary flows were specified in the foreground system. Ref column contains the dataset reference number per Supplementary Information SI2

The pedigree score and processes included in each inventory data are shown in Fig. 2 below.

Figure 2a shows the pedigree scores of the collected inventory data. Completeness, which indicates the duration of data collection from an operating site (e.g., to even out process fluctuations) and number of sites represented, was the lowest scoring category. The most complete unit process dataset was reported for GBFS (Dataset 2.1 per Supplementary Information SI2), in which data from 12 sites were collected over a year. Data collection duration and number of sites represented were not specified in 19 out of 36 inventory data, primarily due to inventory data that were determined from a model or calculation instead of operational measurement. Temporal representativeness was also a low-scoring category, with 20 out of the 36 inventory datapoints being >15 years old. Studies of common clinker substitutes (e.g., GBFS, fly ash) rely on such potentially outdated data, which no longer meet EPD requirements per EN 15804, which states that generic data used must be <10 years old. High reliability (pedigree score of 1 or 2) verified data from operating plant measurements were found for GBFS, limestone, natural pozzolans, recycled concretes, and steel slag carbonation. Newer technologies had more sources of inventory data available in the literature (e.g., calcined clays, and biomass ash). However, some of these data describe lab-scale processes (e.g., in (Salvi Malacarne et al., 2021; Vargas et al., 2020)), which should generally be upscaled to industrial-scale production to improve accuracy in LCA (Tsoy et al., 2020). Furthermore, for calcined clays there were no inventory data available based on verified facility operating data. As of 2023, there were 14 operational calcined clay facilities globally (Perilli, 2025). Verified operational data is required for a data reliability score of ‘good’ or better, and should be collected as a priority for calcined clay.

Figure 2b shows that drying, calcination, quarrying (mineral extraction), milling, and crushing are the most frequently described unit processes in the reviewed literature, each with >3 unit process datasets recorded. Several processes were described only by a single unit process dataset, such as production of RCF (e.g., advanced dry recovery process and heated air classification (Zhang et al., 2019)), carbonation of RCF to produce cRCF (e.g., (Driver et al., 2024)), and specialty slag processing (metal recovery, hot braising, and slag carbonation (Z. Li et al., 2024)). Hence, collecting or creating additional data for these unit processes would be especially beneficial to improve their reliability and generalisability.

Less than half of the unit process datasets included elementary flows in the foreground (Fig. 2b). PM emissions were the most frequently included foreground elementary flow, which is expected for physically processed minerals (European Environment Agency, 2023). However, no PM emissions were reported for the recycling of concrete or beneficiation of biomass ash unit process datasets, which all use physical processing such as crushing or milling. Where elementary flows are not specified in foreground systems, impact assessment is completely reliant on background data (e.g., energy source, electricity grid). This is problematic because LCA results may be underestimated and dependent on the background data selected, which may not be appropriately interrogated in the study. However, PM emissions are not used to calculate climate change impact and, therefore, their omission from the foreground only affects the relevant impact categories (e.g., PM formation potential, impacts to human health).

No elementary flows of heavy metals were reported in the foreground unit process datasets. This is potentially inconsistent with heavy metal presence in mine tailings (Okewale & Grobler, 2023), bauxite residue (Sun et al., 2019), and raw clays (Jurković et al., 2021). Heavy metals present in these materials could be emitted during high temperature (e.g., if volatile) and wet (e.g., in wastewater) processing, or incorporated into the final product. Heavy metals are relevant to impact categories related to toxicity (e.g., ecotoxicity, human toxicity), which is of significant interest for products like cement and concrete since they are dispersed throughout the built environment. More data on the raw material composition into each processing stage, and therefore the expected transformation of chemical species within the unit process, would assist in estimating the likely elementary flows of materials such as heavy metals.

Finally, potential greenhouse gas emissions from raw materials are particularly relevant for assessing the carbon footprint of high-temperature processes such as calcination (Hanein et al., 2021). For example, there is interest in producing calcined clays from low-grade clays which can contain significant sulphate and carbonate content (e.g., UK clays show sulphate and carbonate contents of up to 8.7 wt.% and 55.8 wt.% respectively (A. S. Basavaraj, personal communication, April 28, 2025)). However, no inventory data collected for calcined clay included volatile elementary flows except for water vapour, and therefore the potential impacts of CO_2_ emissions from processed carbonates may be overlooked. Therefore, more complete characterisation of raw materials associated with unit process datasets could assist in more accurately characterising their emissions and improving confidence in the foreground systems.

## Application of the inventory data

### LCA results per process

**Fig. 3:**
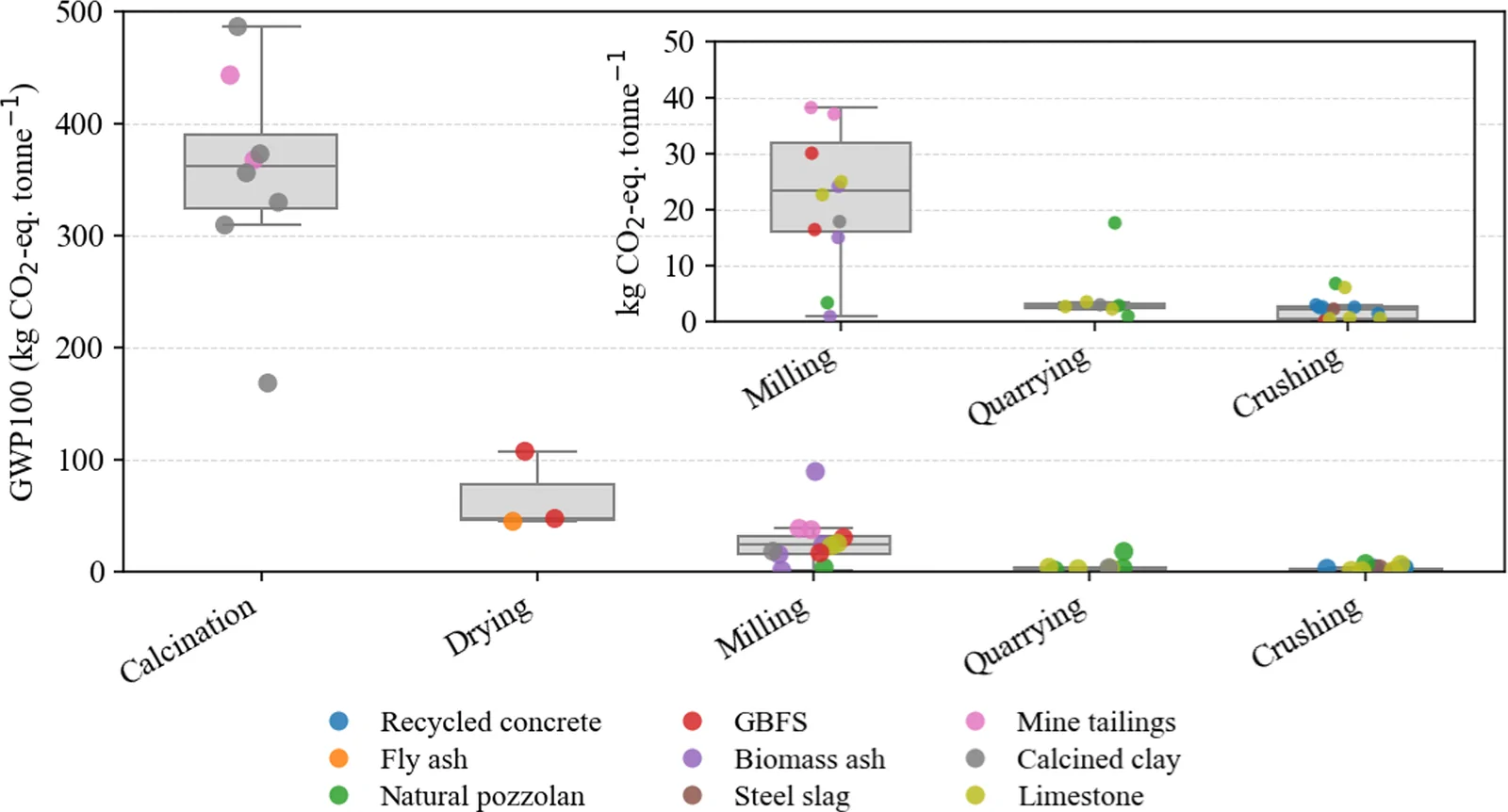
Gate-to-gate carbon footprint of frequently occurring unit processes (calcination, drying, milling, quarrying and crushing) for processing clinker substitutes. Inset used to improve visualisation of processes with median GWP100 <50 kg CO_2_-eq. tonne^—1^. The horizontal line inside the boxes indicates median values. The lower box border indicates the lower quartile (25th percentile), and the upper box border indicates upper quartile (75th percentile). Error bars extend to 1.5 times the interquartile range. Dot points represent individual unit process datasets, coloured by material per the legend

Figure 3 shows the GWP per tonne of frequently (> 3) occurring unit processes in the collected inventory data: calcination, drying, milling, quarrying, and crushing.

Figure 3 shows that calcination and drying, both thermal processes, had the highest carbon footprints with median GWP100 values of ~370 and ~50 kg CO_2_-eq. per tonne product, respectively, primarily due to their fuel requirements for heat. Physical processes (e.g., milling, quarrying and crushing) had the lowest carbon footprints. Both milling and crushing are size reduction processes, with milling having a higher carbon footprint than crushing. This is expected based on Bond work index (Bond, 1955), where energy required for size reduction increases as particle size decreases. Milling generally had a carbon footprint between 17 – 32 kg CO_2_-eq. tonne^—1^ of material processed, when calculated using the European market for electricity. The maximum carbon footprint for milling was ~90 kg CO_2_-eq. tonne^—1^, based on a unit process dataset for milling biomass ash which had an electricity consumption of ~250 kWh tonne^—1^. While 250 kWh tonne^—1^ is an outlier for grinding electricity consumption compared to the other collected datasets, this illustrates the potential uncertainty associated with using individual secondary inventory data, particularly when broader data collection or cross-checking is not performed. Refer to Supplementary Information SI1, S6 Figures S6 and S7 for electricity demand and non-electricity energy demand per process.

These results suggest that mechanical processing, such as grinding and mechanical activation, to improve the reactivity of clinker substitutes are a lower impact processing route than thermal processes such as calcination. While reducing particle size is known to increase the reactivity of clinker substitutes (e.g., Ecocem Superfine, which is a fine grade GBFS product which has comparable performance with silica fume in cement (ecocem, 2026)), the size and distribution of cement particles have a large influence on its performance (e.g., such as hydration kinetics, microstructure development [46]). Smaller particle sizes and higher specific surface area both contribute to increasing the water demand required for workability, which affects the compressive strength of the concrete (Juenger & Siddique, 2015; Pedro Cesar R. A. Abrão et al., 2020). Particle size optimisation of the clinker fraction in cement is emerging as an important method to improve binder reactivity and further reduce clinker factor (e.g., Cemex, 2024; Nieświec et al., 2026; Shi et al., 2021)), notably under investigation by Cemex who report that ‘clinker micronization’ has the potential to reduce clinker factor in cement up to 50% (Cemex, 2024). Increasing the clinker concentration in the fine fraction is reported to be more effective at improving reactivity and allows a higher proportion of inert fillers (e.g., limestone) to be used in the cement without significant reduction in compressive strength performance (Ben Haha et al., 2023). Therefore, while mechanical processing is a lower impact method for producing clinker substitutes, pragmatic improvement of clinker substitute reactivity through particle size reduction is recommended: size reduction must be carefully considered in the context of the overall composite cement performance (e.g., including packing density and water demand) and prioritising clinker content reduction.

Finally, Fig. 3 shows that the calculated carbon footprints for a given unit process generally fell within the interquartile range of all calculated carbon footprints for that process. This suggests that the carbon footprints of unit processes are comparable between different materials (e.g., the carbon footprint of calcined mine tailings and calcined clays are similar). This increases the confidence of applying process-based proxy data for emerging clinker substitutes, as our results can likely be generalised to a wider set of materials, and allowing faster estimation of the carbon footprints of new materials processed using conventional methods for clinker substitutes.

### LCA results per clinker substitute

**Fig. 4:**
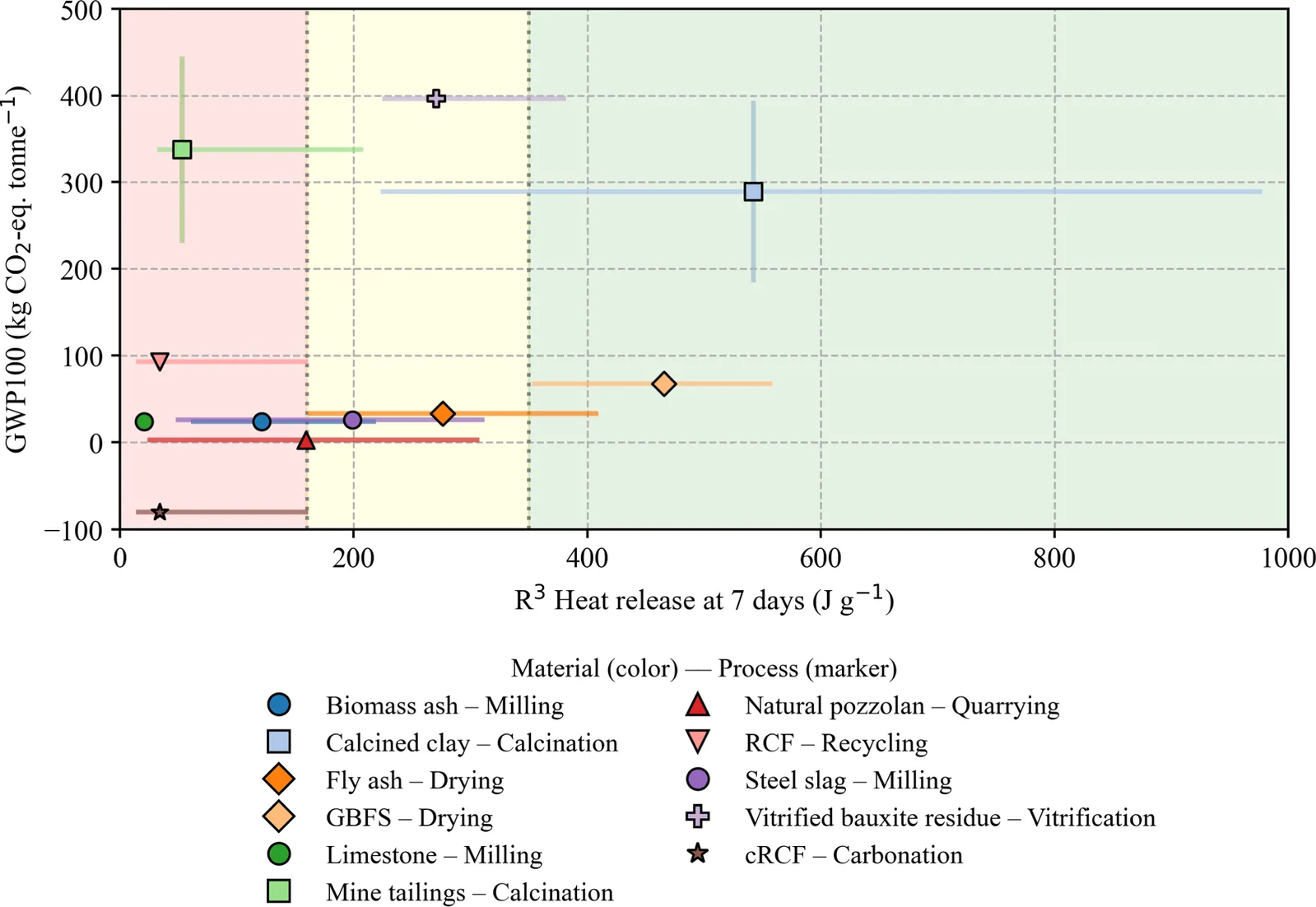
Cradle-to-gate carbon footprint for each material against reported R^3^ heat release reactivity data (Sect. 2.6). Horizontal lines represent the range of reported R^3^ heat release data, and vertical lines represent the range of GWP100 carbon footprint calculated from the RDF (net, lower limit) and coal (upper limit) scenarios (refer to Supplementary Information SI1, S5 Figure S5). Markers are placed horizontally at the median reported R^3^ heat release data, and vertically at the average GWP100 carbon footprint, and marker shapes for each material are based on the unit process with the highest contribution to material GWP100. Red region represents ‘low’ reactivity pozzolans, yellow region represents ‘moderate’ reactivity pozzolans, and green region represents ‘high’ reactivity pozzolans per Sect. 2.6 from data in (Londono-Zuluaga et al., 2022)

All studied clinker substitutes had lower carbon footprints than the average European clinker (~ 832.8 kg CO_2_-eq. tonne^—1^ per Sect. 4.5), and therefore all materials reduce the carbon footprint of composite cements when used as clinker substitutes. However, understanding the material reactivity in addition to material carbon footprint is essential to identifying high potential clinker substitutes, as more reactive clinker substitutes can likely displace more clinker in composite cements. Figure 4 compares the cradle-to-gate carbon footprint of processing clinker substitutes against their reported reactivity.

Based on R^3^ heat release data, calcined clays are the most reactive clinker substitute but are also one of the highest carbon footprint clinker substitutes. We conducted sensitivity analysis on the fuel source providing process heat (Supplementary Information SI1, Section S5 and Figure S5), which demonstrates the decarbonisation potential for higher carbon footprint processes like calcination. Additionally, the contribution of calcined clay to the carbon footprint of composite cement is reduced when used in a tertiary composite cement such as limestone calcined clay, which typically has a 2:1 ratio of calcined clay to limestone (Scrivener et al., 2018a, 2018b). Finally, clay resources suitable for calcined clay production are widely available and generally in proximity to cement plants (Scrivener et al., 2018a, 2018b), and thus are likely to have low transport related emissions and high potential market share with respect to cement demand.

However, calcination is not a global method to producing highly reactive clinker substitutes, as calcined mine tailings had a median R^3^ heat release of ~100 J g^—1^, which is below the 160 J g^—1^ threshold for ‘moderate’ reactivity clinker substitutes. This demonstrates that while processing can increase reactivity, not all intensively processed materials have high reactivity. However, we note that there are other potentially positive effects on cement hydration of using poorly reactive materials in cement (e.g., nucleation). Additionally, other environmental drivers such as reducing mine storage risks and rehabilitation of tailings dams may therefore drive the uptake of mine tailings in cement. Given the low reactivity improvement from calcination, processing methods with lower carbon footprint (e.g., milling) for mine tailings should therefore be investigated to improve the environmental performance of mine tailings as a clinker substitute. Alternatively, vitrification may be an attractive processing method for suitable mine tailings (e.g., bauxite residue), as vitrified bauxite residue has moderate reactivity. However, the decarbonisation potential of vitrification is lower than calcination due to the emissions of CO_2_ from limestone and petroleum coke during the vitrification process.

Uncarbonated RCF has the second-highest carbon footprint of the clinker substitutes with low reactivity (< 160 J g^—1^). The relatively high carbon footprint of the RCF product is related to its low yield from concrete demolition waste, and is dependent on intended market price relative to the price of recycled aggregates (e.g., which can be purchased for £5 – 12 tonne^—1^ in the UK (Dorton Recycling Limited, 2020)). As RCF is not yet commercially available, our analysis (Fig. 4) assumes an RCF price of £100 tonne^—1^, comparable with bulk CEM I supply in the UK. If RCF is instead offered at a price comparable with recycled aggregates (e.g., £12 tonne^—1^), the carbon footprint reduces to ~40 kg CO_2_-eq. tonne^—1^, resulting in a carbon footprint comparable with lower-impact low reactivity clinker substitutes such as biomass ash and limestone.

The only carbon-negative clinker substitute investigated here is cRCF, due to the mineralisation of CO_2_ to produce calcium carbonates from the uncarbonated material (RCF). While there are no published R^3^ data for cRCF, we expect similar reactivity to RCF (< 160 J g^—1^) (Sousa et al., 2024) based on preliminary results in ongoing research (S. Sreeram, personal communication, August 13, 2025). There is also growing interest in using cRCF as a limestone replacement in calcined clay blends (e.g., Baki et al., 2026; Liu et al., 2025; Wang et al., 2025)), which would improve their utility as a clinker substitute despite its otherwise low reactivity. The carbonation process modelled here is a semi-dry process which uses hot flue gas from clinker production, and therefore requires no external energy for heating. It assumes that 1 tonne of raw RCF could mineralise ~210 kg CO_2_ (Driver et al., 2024), whereas mineralisation potentials reported elsewhere are lower at 40 – 180 kg CO_2_ tonne^—1^ RCF (Heidelberg Materials, 2024; Nedunuri et al., 2021; Tabrizi et al., 2025). The carbon footprint of cRCF would increase for applications which require energy input (e.g., which if approximated using the carbon footprint of drying in Fig. 3 could increase it by 50 – 100 kg CO_2_-eq. tonne^—1^), and if the raw fines had a lower CO_2_ mineralisation potential. The mineralisation potential of the RCF is dependent on its composition, which is impacted by the original concrete mix design, CO_2_ uptake during its upstream life cycle, and separation technology used (e.g., which affects the yield and composition of the separated fine fraction (Carriço et al., 2021)). Additionally, our analysis did not consider transport related emissions, which are expected to be significant for cRCF as it must be transported from the demolition site to the separation processing site, and then to the flue gas source which is likely to be in industrial rather than urban area. The data presented here, therefore, likely represents the ‘best-case’ scenario for carbonation of RCF. Investigating the carbon footprint of cRCF under more scenarios (e.g., lower boundary of mineralisation potential, high transport distances, non-flue gas CO_2_ source) would therefore be valuable in fully establishing its viability to reduce global cement industry emissions.

## Discussion

### Environmental performance of composite cements

Table 3 shows the processing carbon footprints of composite cements per EN 197–1, and the relative contributions of clinker and clinker substitutes, per tonne of cement produced. Note that the carbon footprint of clinker can vary based on processing conditions and fuel type, and the values used for clinker in this assessment are based on reported industry average for European production (refer to Supplementary Information SI1, Section S4).

**Table 3:**
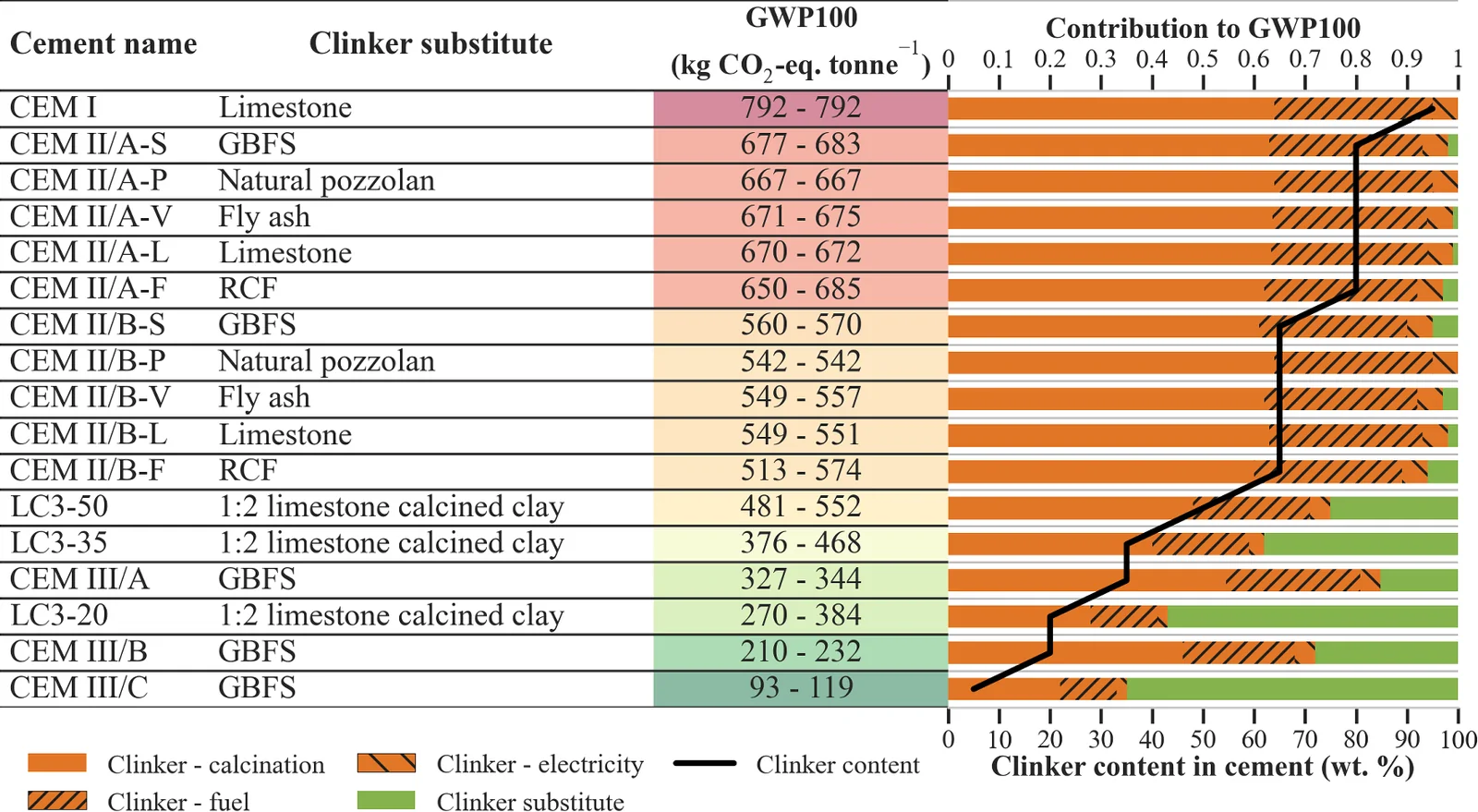
Carbon footprint of select composite cements per EN 197 and LC3-20, calculated per Eq. (1). Note that other composite cements, including ternary cements other than LC3, are also available per EN 197. Transport emissions were excluded (e.g., d_t_ = 0). The lower GWP100 values are for clinker substitute production using RDF fired heat; the upper GWP100 values are for clinker substitute production using coal fired heat. For RCF, we considered the use of cRCF to determine the lower GWP100, and uncarbonated RCF as the upper GWP100. The colour gradient is from worst (red) to best (green). The visualised contribution analysis is based on the upper GWP100 scenario: Orange represents total clinker emissions, broken down by clinker calcination contribution (solid orange), clinker fuel contribution (upwards diagonal orange), and clinker electricity contribution (downwards diagonal orange). Solid green represents contributions from the clinker substitute. The black line represents the clinker factor in cement. The basis of this comparison is per unit mass of cement produced, which does not imply functional equivalence (e.g., compressive strength performance) between blends

Overall, Table 3 shows that the composite cements with the lowest clinker content had the lowest carbon footprint, suggesting that reducing clinker factor has a greater effect on composite cement emissions than clinker substitute type. For example, clinker substitutes like calcined clay, which have a higher carbon footprint on a mass basis (Fig. 4), can produce composite cements with lower carbon footprint than other less intensively processed clinker substitutes (e.g., natural pozzolans, fly ash), due to the lower clinker content. In LC3-50, lower clinker factor is supported by the high reactivity of calcined clay, with LC3-50 cements achieving comparable 28-day strength performance to CEM I cements and therefore suitable for general purpose use (Scrivener et al., 2018a, 2018b). The use of cRCF in CEM II/B-F (i.e., lower GWP100 value) reduced emissions by ~30 – 45 kg CO_2_-eq. tonne^—1^ cement compared to other clinker substitutes (limestone, natural pozzolan, fly ash) in composite cements with 65 wt.% clinker. However, LC3 cements may have lower carbon footprint than cRCF-containing CEM II cements, depending on the composition and processing requirements of the each system, despite the negative carbon footprint of the cRCF.

The LC3 cements were the lowest emission cements except for CEM III/A-C, which have very low clinker factor (< 35 wt.% clinker in cement). Beyond carbon footprint, cement mix design is also driven by the availability, price, and required performance of the concretes. CEM III/A-C cements cannot be produced in sufficient quantities to meet the annual global cement demand of ~4100 Mt (U.S Geological Survey, 2024), as there is only 330 – 410 Mt of GBFS available annually which is almost fully utilised as in cement (Edwards et al., 2026). Additionally, as this analysis considered processing only carbon footprint, the results for GBFS and CEM III cements do not include upstream allocation from pig iron production. With economic allocation, we estimate the carbon footprint allocated to GBFS could increase by up to ~480 kg CO_2_ tonne^—1^ GBFS due to high GBFS prices (up to £160 tonne^—1^ GBFS, refer to Supplementary Information SI1, Section S3.2). We note that this value illustrates the potential influence of economic allocation which must use the most applicable price data available for product-specific LCA, rather than a universally applicable carbon footprint for GBFS. Finally, our analysis compares cements on a mass basis only to demonstrate the utility of the inventory data collected in this study, and does not assess the technical performance of or imply functional equivalence between composite cements. For example, slower strength formation has been reported at high GBFS content in cement (e.g., reported experimental results show that 28-day strength decreases with >40 wt.% GBFS content in cement Bilim et al., 2009; Kumar et al., 2015)), although in regions where GBFS is widely available CEM III/A cements are widely used as general purpose concretes. The future availability of GBFS may nevertheless affect the extent to which these cements can be deployed for deep decarbonisation of the cement sector. Combining the inventory data presented here with performance (e.g., compressive strength, durability) of low clinker cements could identify the most promising low environmental impact, high technical performance cement mix designs (e.g., kg CO_2_ m^—3^ concrete MPa^—1^ compressive strength).

CEM III/C and LC3-20 cements were the only composite cements where the clinker substitute processing had the highest contribution to the carbon footprint of cement instead of clinker. For composite cements where clinker overall had the highest contribution, clinker calcination emissions had the highest contribution to total carbon footprint. In the context of producing composite cements aligned with the GCCA decarbonisation roadmap, which targets average clinker content in cement of 58 wt.% and 52 wt.% by 2030 and 2050 respectively (GCCA, 2021), clinker will still be the dominant contribution to composite cement emissions irrespective of clinker substitute used. Therefore, future research efforts should prioritise efficient use of clinker in cement and increasing clinker reactivity.

## Conclusion

We collected 36 sets of inventory data for processing of the most important clinker substitutes, which were disaggregated into 57 unit processes datasets. The most frequently recorded processes were calcination, heat-fired drying, quarry extraction, crushing, and milling, each recorded in >3 unit process datasets. However, data completeness and temporal representativeness both had overall low scores across the collected inventories, for example finding that ~55% of data were >15 years old. The priorities for further inventory data development are: (1) developing an inventory of calcined clay production unit processes from operating plant data, (2) collecting more data for specialty processes (e.g., RCF and carbonation), and (3) developing a methodology to incorporate raw material composition into inventory data to better characterise foreground elementary flows (e.g., for volatile processes).

We found that calcination had the highest carbon footprint (median ~370 kg CO_2_-eq. per tonne) of the processes considered. Mechanical processes, such as crushing and milling, were lower impact (generally <35 kg CO_2_-eq. per tonne). However, the energy demand of mechanical processes generally increases as particle size decreases, and improvements in clinker substitute reactivity associated with mechanical treatments which reduce particle size must be balanced against the potential effects of increasing the fine fraction on concrete performance. Lower impact mechanical processes are likely a preferred processing method for materials like mine tailings, which have low reactivity but a high carbon footprint when calcined. Therefore, this work can inform technology development and business decisions related to the concrete value chain, improve the transparency of LCA studies of concrete materials, and act as a baseline to verify commercial data.

## Supplementary Information

Below is the link to the electronic supplementary material.

Supplementary file1 (DOCX 696 KB)

Supplementary file2 (XLSX 924 KB)

## Data Availability

No datasets were generated or analysed during the current study.

## Summaries

Supplementary Information SI1 contains material descriptions and additional information for LCA (detailed economic allocation results for RCF and GBFS, calculated impacts for European clinker, and justification for fuel sensitivity analysis).

Supplementary Information SI2 contains the collected inventory data as a repository of unit process diagrams. Supplementary Information SI2 also includes pedigree scores, summary data and default values for energy unit conversions.
