# Supplementary material for: Toward process-based inventory data for Portland clinker substitutes
Source: J Ind Ecol. 2026 Sep 18;30(5):2661–77. doi: 10.1007/s44498-026-00186-x (PMC13649849; doi:10.1007/s44498-026-00186-x)
Supplement: Supplementary file 1 [file 44498_2026_186_MOESM1_ESM.docx]

**Supplementary Information SI1: Toward Process-Based Inventory Data for Portland Clinker Substitutes**

Pippa Edwards^1,a^, Wilson Ricardo Leal da Silva^2,b^, Paul Fennell^3,c^, Rupert J. Myers^1,d,^*

^1^ Department of Civil and Environmental Engineering, Imperial College London, SW7 2AZ, London, UK

^2^ Fuller Technologies, Copenhagen, DK

^3^ Department of Chemical Engineering, Imperial College London, SW7 2AZ, London, UK

*Corresponding author. Email: ^a^ [pippa.edwards@imperial.ac.uk](mailto:pippa.edwards@imperial.ac.uk); ^b^ [wld@fullertechnologies.com](mailto:wld@flsmidth.com); ^c^ [p.fennell@imperial.ac.uk](mailto:p.fennell@imperial.ac.uk); ^d^ [r.myers@imperial.ac.uk](mailto:r.myers@imperial.ac.uk)

Supplementary Information SI1 contains material descriptions and additional information for LCA (detailed economic allocation results for RCF and GBFS, calculated impacts for European clinker, and justification for fuel sensitivity analysis).

Table of Contents

[S1 Data pedigree	2](#_Toc238115432)

[S2 Process overview for clinker substitutes	3](#_Toc238115433)

[S2.1 Fly ash	3](#_Toc238115434)

[S2.2 GBFS	3](#_Toc238115435)

[S2.3 Calcined clay	3](#_Toc238115436)

[S2.4 Limestone	4](#_Toc238115437)

[S2.5 Natural pozzolans	4](#_Toc238115438)

[S2.6 Recycled concrete	4](#_Toc238115439)

[S2.7 Biomass ash	4](#_Toc238115440)

[S2.8 Steel slag	5](#_Toc238115441)

[S2.9 Bauxite residue	5](#_Toc238115442)

[S2.10 Mine tailings	5](#_Toc238115443)

[S3 Economic allocation	6](#_Toc238115444)

[S3.1 Recycled concrete fines	6](#_Toc238115445)

[S3.2 Upstream allocation of pig iron production to GBFS	6](#_Toc238115446)

[S4 European clinker inventory	8](#_Toc238115447)

[S5 Contribution analysis for conventional clinker substitutes	10](#_Toc238115448)

[S6 Electricity and non-electric energy demand per unit process	13](#_Toc238115449)

[S7 References in this supporting information file	14](#_Toc238115450)

# S1 Data pedigree

The data pedigree matrix proposed by Weidema and Wesnaes (Weidema & Wesnæs, 1996) is presented for reference below in Table S1.

Table S1: Data quality pedigree matrix (adapted from (Weidema & Wesnæs, 1996))

| **Indicator** | **1 (Strongest)** | **2** | **3** | **4** | **5 (Weakest)** |
| --- | --- | --- | --- | --- | --- |
| Reliability (R) | Verified data based on measurements | Verified data partly based on assumptions or non-verified measured data | Non-verified data partly based on assumption | Qualified estimate (e.g., by industrial expert) | Non-qualified estimate |
| Complete-ness  (C) | Representative data from a sufficient sample of sites over an adequate period to even out normal fluctuations | Representative data from a smaller number of sites but for adequate periods | Representative data from an adequate number of sites but from shorter periods | Representative data from a smaller number of sites/shorter periods or incomplete data from adequate number of sites | Representativeness unknown or incomplete data from a smaller number of sites and/or from shorter periods |
| Temporal correlation  (T) | <3 years difference to year of study | <6 years difference | <10 years difference | <15 years difference | Age unknown or >15 years difference |
| Geographic correlation  (G) | Data from area under study | Average data from larger area in which the study area is included | Data from area with similar production conditions | Data from area with slightly similar production conditions | Data from unknown area of area with different production conditions |
| Further technology correlation  (F) | Data from enterprises, processes, and materials under study | Data from processes and materials under study but from different enterprises | Data from processes and materials under study but from different technology | Data on related processes or materials but same technology | Data on related processes or materials but different technology |

# S2 Process overview for clinker substitutes

## S2.1 Fly ash

Fly ash is produced as a by-product of coal-fired electricity, where it is collected from the combustion process (e.g., from electrostatic precipitators or bag filters). Unless used directly, fly-ash may be stockpiled prior to preparation for use in cement. Fly ash reactivity has been extensively investigated, with 7-day heat release results per R3 test between 160 – 409 J g^—1^ (Al-Shmaisani et al., 2022; Frías et al., 2024; Koufany et al., 2025; Li et al., 2018; Londono-Zuluaga et al., 2022; Snellings & Scrivener, 2016; Suraneni et al., 2019).

Of the reviewed studies involving fly ash, 22 studies used only an impact category indicator value for fly ash (e.g., total CO_2_-eq. per unit mass) without reporting an inventory for fly ash processing. A single inventory dataset was available in Chen et al. (2009) (Chen et al., 2010) based on data from French producer Surschiste (Surchiste, 2021), describing the drying of stockpiled fly ash as part of a reclamation process. This data has been replicated in other studies (e.g., (A. P. Gursel, 2014)) and tools (e.g., (A. Gursel, 2012)). The fly-ash reclamation process operated by Surschiste involves (Surchiste, 2021):

1. Collection of fly ash from power plants (dust collector unit – e.g., electrostatic precipitator); and
2. Storage of ash in silo; or
3. Wetting and storage of ash in a stockpile (presumably wetted for dust control); and
4. Drying of wetted stockpiled ash per client requirement using dryer systems with capacity between 50-80 tph.

## S2.2 GBFS

Ground granulated blast furnace slag is produced using molten slag from pig iron production, which is rapidly quenched to preserve amorphous phase structure prior to being dewatered, dried and ground for use in cement. GBFS is considered a reactive clinker substitute, with 7-day heat release results per the R3 test between 350 – 560 J g^—1^ (Al-Shmaisani et al., 2022; Li et al., 2018; Londono-Zuluaga et al., 2022; Snellings & Scrivener, 2016; Suraneni et al., 2019).

We reviewed 89 studies were reviewed for inventory data pertaining to GBFS processing. A total of 9 studies used unclear or unavailable source data, 20 studies reported an impact factor only (e.g., embodied GWP, embodied CO_2_, and/or embodied energy), and 3 assumed GBFS processing was burden-free. 63 of the reviewed articles had clear source data for GBFS impact assessment (whether LCA or impact factors). The most prominent source of data was a 2003 survey of twelve operating sites in the United States originally presented in a report titled “Life Cycle Inventory of Slag Cement Manufacturing Process” (Marceau & VanGeem, 2003). This study is the source data of multiple prominent works and databases with GBFS inventory data (e.g., (Chen et al., 2010; A. P. Gursel, 2014; Kellenberger et al., 2007)), and was used in at least 41 of the reviewed studies, representing 65% of the work with clear source data.

## S2.3 Calcined clay

Calcined clay suitable for use as a clinker substitute can be produced from common clay with a minimum 40% kaolinite content. The clay is calcined to improve reactivity, by heating to the dehydroxylation temperature (typically 650-850 °C) to deform kaolinite into metakaolin (Scrivener et al., 2018). The reactivity of calcined clay is dependent on the kaolinite content, with measured 7-day heat release between 220 – 980 J g^—1^ (Al-Shmaisani et al., 2022; Frías et al., 2024; Koufany et al., 2025; Li et al., 2018; Londono-Zuluaga et al., 2022; Suraneni et al., 2019). Calcined clays have been shown to have comparable mechanical performance to conventional Portland cement after 7 days when mixed with clinker (50%), limestone (15%), and gypsum (5%) in an ‘LC3’ blend (30% calcined clay) (Scrivener et al., 2018).

Of the 31 studies identified assessing calcined clay, 8 studies used a pre-aggregated emission factor for calcined clays as inventory data. Four publicly available sources of LCI data were identified from the studies reviewed. An additional two datasets were added based on the authors’ awareness of the literature.

## S2.4 Limestone

Limestone is a common minor mineral addition to cement, in addition to use as a primary raw material for Portland cement clinker. It is extracted from a limestone quarry, which can require explosives, before being crushed and milled for use in cement. Limestone is poorly reactive in cement, with 7-day heat release measured as low as 21 J g^—1^ (Suraneni et al., 2019), however it is both a suitable filler material (which can be used up to ~15 wt.% in cement to promote nucleation), and improves the performance of calcined clay in LC3 cement blends (Scrivener et al., 2018).

## S2.5 Natural pozzolans

Natural pozzolans describe a range of naturally occurring materials, typically high in silica and produced from volcanic rock or sedimentary deposits of diatomaceous earth. Similarly to limestone, natural pozzolans are quarried, crushed and milled for use in cement, but are typically not further processed. The reactivity of natural pozzolans varies depending on the material type, and 7-day heat release results have been measured between 24 – 310 J g^—1^ (Al-Shmaisani et al., 2022; Frías et al., 2024; Koufany et al., 2025; Li et al., 2018; Londono-Zuluaga et al., 2022; Snellings & Scrivener, 2016; Suraneni et al., 2019).

## S2.6 Recycled concrete

Concrete demolition waste can be recycled into useful concrete products, including recycled concrete aggregates, recycled concrete fines, and carbonated recycled concrete fines. Recycled concrete aggregates are produced from crushing concrete demolition waste into various size fractions of aggregate, and additional processing (e.g., Advanced Dry Recovery (‘ADR’) and Heated Air Separation (‘HAS’)) can further separate fine cementitious material (recycled concrete fines, RCF) from aggregate and sand. Several sets of primary data were collected for recycled aggregate production from concrete demolition waste, while only a single set of data was available for each of the advanced processing to produce RCF, and carbonated RCF (‘cRCF’).

The RCF product can be further processed by mineral carbonation, where using flue gas containing CO_2_ in a partially wet contact process is a promising process technique currently employed in pilot plants (e.g., Heidelberg Poland, who have estimated that 1 t of cRCF product could sequester 150 kg of CO_2_ (Heidelberg Materials, 2024)). Heat release data for RCF has been measured between 30 – 160 J g^—1^ (Frías et al., 2024; Sousa et al., 2024). Currently, there is a lack of published data for the potential improvement in reactivity from carbonating RCF.

## S2.7 Biomass ash

Biomass ashes are collected from the production of bioenergy, where various sources of biomass are burned in combustion chambers, and suitable ashes can then be milled to improve their pozzolanic reactivity for use in cement. The heat release of biomass ashes have been measured between 60 – 220 J g^—1^ (Frías et al., 2024; Londono-Zuluaga et al., 2022; Suraneni et al., 2019). All inventory data relating to biomass ash processing involved milling only.

## S2.8 Steel slag

Slags from steelmaking are less commonly used as clinker substitutes than granulated blast furnace slag. Steel slag composition is variable depending on the steel being produced, but is rich in calcium oxide from the lime flux. Steel slag is typically much less reactive than GBFS, with measured 7-day heat-release between 45 – 300 J g^—1^ (Frías et al., 2024; Londono-Zuluaga et al., 2022; Suraneni et al., 2019; Wang & Suraneni, 2019). We collected several up-cycling datasets for steel slag, including hot braising of steel slags to dissolve free CaO and prevent expansion as aggregate, screening and crushing of slags, metal extraction from slag, and steel slag carbonation.

## S2.9 Bauxite residue

Bauxite residue is a waste product from alumina production, and is stored in tailings dams. We collected data for filter pressing residue to remove water, as well as vitrification of bauxite residue with coking coal to improve the reactivity of the product in cement. Raw bauxite residue reactivity has been reported between 80 – 210 J g^—1^ heat release at 7 days (Hertel et al., 2022; Yao et al., 2024), and vitrified or calcined bauxite residue has reported 7-day heat release between 220 – 380 J g^—1^ (Giels et al., 2025; Yao et al., 2024).

## S2.10 Mine tailings

Mine tailings other than bauxite residue have also been investigated for use as clinker substitutes, including gold, copper and lead tailings (Yao et al., 2024). However, calcined mine tailings have generally low reactivity even after heat treatment (7-day heat release between 55 – 95 J g^—1^ for non-bauxite tailings (Yao et al., 2024)), which overlaps with reported reactivity for mechanically activated mine tailings (30 – 130 J g^—1^ (Ramanathan et al., 2021)). Inventory data were available for air-drying, grinding, and calcination of stockpiled mine tailings.

# S3 Economic allocation

## S3.1 Recycled concrete fines

As RCF is not yet commercially available, there are no data to support its price for economic allocation purposes. Figure S1 shows the GWP100 of RCF over RCF price range £0 – 200 tonne^—1^, assuming aggregate prices of £5 tonne^—1^ 0.125 – 4mm RCA, and £12 tonne^—1^ 4 – 12 and 12 – 22 mm RCA.

Figure S1: Economic allocation for RCF as a function of RCF price. Grey vertical band on upper plot demonstrates the comparison CEM 1 price, and pink horizontal band on lower plot represents the range of GWP100 calculated for recycled concrete aggregates (RCA) from other data.

We compared the allocated impact to the RCA products to calculated GWP100 for RCA products without production of RCF, on the basis that the RCA co-products would need to have comparable GWP to be competitive. We additionally compared the RCF price to the estimated CEM I price of £100 – 150 tonne^—1^, as RCF is intended as a cement substitute. The viable price of RCF is therefore bounded by the minimum price for RCA products to have comparable GWP, and the upper price range of CEM 1 cement, which Figure S1 demonstrates is between £50 – 150 tonne^—1^. Based on this analysis, we assumed a price of £100 tonne^—1^ for our analysis, which results in a carbon footprint of ~93 kg CO_2_-eq. tonne^—1^ RCF.

## S3.2 Upstream allocation of pig iron production to GBFS

Pig iron production has a carbon footprint of approximately 1500 kg CO_2_-eq. tonne^—1^ pig iron, with co-production of ~240 kg slag (later processed into GBFS) (Carpenter, 2012; Chen et al., 2010). As GBFS is a useful by-product, the impacts of pig-iron production should be shared between GBFS and pig iron. Previous studies of the economic allocation factors of GBFS (Chen et al., 2010) assumed a slag:pig iron price ratio of 0.1. However, the market price of GBFS can reach up to £160 tonne^—1^ in the UK (Edwards et al., 2025), compared to a 2026 basic pig iron price of £450 – 500 tonne^—1^ (Foundry Trade Journal, 2026), resulting in a price ratio of 0.35. Figure S2 demonstrates the sensitivity of GWP allocated to GBFS from pig iron production to the GBFS:pig iron price ratio.

Figure S2: GWP allocated to GBFS from pig iron production as a function of GBFS:pig iron price ratio. Based on total pig iron GWP of ~1500 kg CO_2_-eq. tonne^—1^ pig iron produced, with 0.24 tonne GBFS co-produced (Chen et al., 2010)

Based on Figure S2, GBFS is allocated ~480 kg CO_2_-eq. tonne^—1^ GBFS at a GBFS:pig iron price ratio of 0.35.

# S4 European clinker inventory

We computed the GWP100 of the clinkers available in ecoinvent, and compared to the GCCA Getting the Numbers Right (GNR) gross CO_2_ emissions data given for each region per Table S2 below. GNR gross CO_2_

We report both the latest GNR data (2021) and the weighted average of the period corresponding to the ecoinvent period.

Table S2: Clinker GWP100 from EcoInvent, against the 2021 reported data from GCCA GNR data for weighted average clinker production for that region (weighted average of all kiln types).

| **ecoinvent flow (data year)** | **GWP100**  **(kg CO_2_-e t^—1^)** | **GNR region** | **2021 GNR CO_2_**  **(kg CO_2_ t^—1^)** |
| --- | --- | --- | --- |
| clinker \| US (2005-2009) | 947.4 | US | 858.4 |
| clinker \| Europe no CH (2005-2009) | 932.1 | EU27 | 790.0 |
| clinker \| CH (2005-2009) | 817.0 |  |  |
| clinker \| IN (2014-2017) | 882.9 | India | 820.7 |
| clinker \| BR (2016) | 929.8 | Brazil | 841.6 |
| clinker \| CO (2014-2017) | 933.9 | Latin America | 840.2 |
| clinker \| RoW (no date) | 942.6 | World | 822.4 |

This shows that the reported 2021 GNR data is lower than the ecoinvent clinkers in all regions, by approximately 50-120 kg CO_2_ t^—1^. However, the GNR data excludes CO_2_ from on-site power production, which may account for some of the difference. Power consumption is reported separately in the GNR data, approx. 80 kWh t^—1^ clinker for the process including upstream of clinker production, and 120 kWh t^—1^ clinker for the full site. Using market for electricity, medium voltage, for Europe as background data from ecoinvent, which has a GWP100 of ~0.36 kg CO_2_ kWh^—1^, the electrical contribution to clinker emissions at the higher end is approx. 42.8 kg CO_2_ t^—1^ ground clinker. Therefore, the carbon footprint of European clinker production can be estimated at 832 kg CO_2_-eq. tonne^—1^ clinker in Europe.

It is possible to create a clinker dataset from the GNR data showing the process (calcination), fuel, and electrical contributions. Thermal energy consumption, power consumption, and carbon intensity of the fuel mix is specified in the GNR data along with the total emissions. Additionally, the total and thermal emissions are also broken down by kiln type.

Table S3: GCCA GNR data for cement kilns with preheater and precalciner (best available technology) for the year 2021.

| **Region** | **Gross CO_2_ emissions excluding onsite power**  **(a)**  **(kg CO_2_ t^—1^ clinker)** | **Thermal energy**  **(b)**  **(MJ t^—1^ clinker)** | **Carbon intensity of the fuel mix**  **(c)**  **(kg CO_2_ MJ^—1^)** |
| --- | --- | --- | --- |
| Cembureau members | 798.3 | 3553.3 | 71.6 |
| EU27 | 788.3 | 3565.8 | 69.3 |
| South/Latin America | 830.2 | 3487.9 | 82.4 |
| World | 819.3 | 3344.9 | 82.7 |
| Austria | 778.3 | 4084.4 | 63.2 |
| Brazil | 852.5 | 3600.6 | 78.8 |
| Czechia | 771.6 | 3691.5 | 63.9 |
| Egypt | 877.7 | 3918.9 | 85.3 |
| France | 783.7 | 3489.8 | 73.6 |
| Germany | 798.6 | 3557.0 | 67.2 |
| India | 819.8 | 3038.0 | 94.3 |
| United States of America | 835.4 | 3555.6 | 81.4 |
| United Kingdom | 841.8 | 3611.8 | 72.6 |

The fuel related emission (in kg CO_2_ t^—1^ clinker) can be calculated by Eqn. (S1) using b and c factors from Table S3:

$fuel emission=b\times c$	(S1)

The process related emission (in kg CO_2_ t^—1^ clinker) can be calculated by Eqn. (S2) using the a factor from Table S3:

$process emission=a-fuel emission$	(S2)

The process emission can be used to estimate the CaO content of the clinker, assuming 100% of the CaO is from carbonate (CaCO_3_) sources, by inverting the IPCC methodology for calcination emissions (IPCC, 2000).

Table S4: Average process and fuel CO_2_ t^—1^ clinker contribution to 2021 GNR data for BAT kiln technology, and estimation of CaO content of the average clinker

| **Region** | **Process contribution**  **(kg CO_2_ t^—1^ clinker)** | **Fuel contribution**  **(kg CO_2_ t^—1^ clinker)** | **Estimated clinker CaO content (wt.%)** |
| --- | --- | --- | --- |
| Cembureau members | 537.9 | 263.1 | 68.5% |
| EU27 | 533.8 | 256.2 | 68.0% |
| South/Latin America | 540.5 | 299.6 | 68.9% |
| World | 536.1 | 286.3 | 68.3% |
| Austria | 526.1 | 254.0 | 67.0% |
| Brazil | 557.0 | 284.5 | 71.0% |
| Czechia | 538.2 | 235.1 | 68.6% |
| Egypt | 547.2 | 332.1 | 69.7% |
| France | 560.5 | 281.5 | 71.4% |
| Germany | 530.2 | 253.3 | 67.5% |
| India | 534.3 | 286.5 | 68.1% |
| United States of America | 551.9 | 306.5 | 70.3% |
| United Kingdom | 558.4 | 279.7 | 71.1% |

The electrical contribution can then be added. This is calculated by multiplying the average cement plant power consumption (kWh t^—1^ cement), approx. 100-120 kWh t^—1^ cement, by the relevant emissions factor for the selected grid.

# S5 Contribution analysis for conventional clinker substitutes

We computed the GWP100 for conventional clinker substitutes (calcined clay, GBFS, limestone, fly ash and natural pozzolan) using process heat from European coal combustion (heat production, at hard coal industrial furnace 1-10 MW) to replicate conditions for conventional clinker production, using the inventories described in the main text. The process contributions to GWP100 are shown in Figure S3.

Figure S3: Process contributions to GWP100 from initial GWP calculation for cement substitutes, with coal-fired heat source (1-10MW)

Figure S3 shows the largest contributor towards carbon footprint was coal derived process heat. This is confirmed by the percentage contributions, shown in Figure S4, excluding the natural pozzolan process where the major contribution was diesel (quarry excavation) and did not have any heat driven process associated with its production.

Figure S4: Relative share of GWP100 contribution for each material.

Therefore, a sensitivity analysis under different fuel scenarios was performed. Figure S5 shows the cradle-to-gate carbon footprint of materials processed for use as clinker substitutes, under the three scenarios for fuel supplying process heat.

Figure S5: Total cradle-to-gate carbon footprint of materials processed for use as clinker substitutes, under three fuel scenarios for process heat: coal is blue, natural gas is orange, and RDF (net) is green. (Net) refers to the non-biogenic CO_2_-eq. emissions associated with RDF fuel use. The diagonal green line series represents the biogenic component associated with RDF fuel use. Inset used to improve visualisation of processes with GWP100 <50 kg CO_2_-eq. tonne.

All studied clinker substitutes had lower carbon footprints than the average European clinker (~832.8 kg CO_2_-eq. tonne^—1^ per Section 4.5), and therefore all materials reduce the carbon footprint of composite cements when used as clinker substitutes. Calcined materials (mine tailings, and clay), have the highest carbon footprints between 184 – 444 kg CO_2_-eq. tonne^—1^ depending on the fuel used, or ~20 – 50% of European clinker carbon footprint. Only natural pozzolans had a carbon footprint below 1% of clinker emissions, a typical data cut-off in LCA. Therefore, with these inventory data now being more accessible through this paper, it is generally not appropriate to assume clinker substitute emissions have negligible contribution to the carbon footprint of composite cements.

Products using natural gas as the source of process heat had the lowest gross carbon footprint, however when considering fossil-derived CO_2_-eq. emissions only, the RDF scenario resulted in lower carbon footprint products. Both the natural gas and gross RDF fuel scenarios resulted in lower carbon footprint materials than the coal scenario. However, under the lowest impact scenarios, calcined materials (calcined mine tailings, calcined clay) still had order of magnitude higher carbon footprints than mechanically processed materials (e.g., limestone, steel slags, biomass ash, and natural pozzolans), which demonstrates that while there is significant decarbonisation potential of high impact processes like calcination, less intensive processing methods should be prioritised where possible.

# S6 Electricity and non-electric energy demand per unit process

Figure S6: Gate-to-gate electricity demand (kWh) of frequently occurring unit processes (calcination, drying, milling, quarrying and crushing) for processing clinker substitutes. Inset used to improve visualisation of processes with median electricity demand <20 kWh tonne^—1^. The horizontal line inside the boxes indicates median values. The lower box border indicates the lower quartile (25th percentile), and the upper box border indicates upper quartile (75th percentile). Error bars extend to 1.5 times the interquartile range. Dot points represent individual unit process datasets, coloured by material per the legend.

Figure S7: Gate-to-gate non-electrical energy demand (MJ) of frequently occurring unit processes (calcination, drying, milling, quarrying and crushing) for processing clinker substitutes. Inset used to improve visualisation of processes with median electricity demand <20 kWh tonne^—1^. The horizontal line inside the boxes indicates median values. The lower box border indicates the lower quartile (25th percentile), and the upper box border indicates upper quartile (75th percentile). Error bars extend to 1.5 times the interquartile range. Dot points represent individual unit process datasets, coloured by material per the legend.

# S7 References in this supporting information file

Al-Shmaisani, S., Kalina, R. D., Ferron, R. D., & Juenger, M. C. G. (2022). Comparison of SCM reactivity to performance in cement-based mixtures. Materials and Structures, 55(10), 241. https://doi.org/10.1617/s11527-022-02072-x

Carpenter, A. (2012). CO_2_ abatement in the iron and steel industry. https://usea.org/sites/default/files/012012_CO2%20abatement%20in%20the%20iron%20and%20steel%20industry_ccc193.pdf

Chen, C., Habert, G., Bouzidi, Y., Jullien, A., & Ventura, A. (2010). LCA allocation procedure used as an incitative method for waste recycling: An application to mineral additions in concrete. Resources, Conservation and Recycling, 54(12), 1231–1240. https://doi.org/10.1016/j.resconrec.2010.04.001

Edwards, P., Leal da Silva, W. R., Fennell, P., & Myers, R. J. (2025). Performance of alternative Portland clinker feedstocks and substitutes: A multi-criteria evaluation. (Under Review).

Foundry Trade Journal. (2026, January 30). Metal Prices. https://www.foundrytradejournal.com/news/metal-prices

Frías, M., Alujas, A., Moreno-Reyes, A., Ibañez, J. A., Paredes, M. V., & Vegas, I. (2024). Advances in understanding R3 chemical reactivity in various traditional and emerging pozzolans: Chemical, mineralogical and calorimetric dimensions. Construction and Building Materials, 457, 139474. https://doi.org/10.1016/j.conbuildmat.2024.139474

Giels, M., Hertel, T., Gamaletsos, P. N., Hallet, V., & Pontikes, Y. (2025). Reactive precursors for inorganic polymers and blended cements from vitrified bauxite residue. A case study for Europe. Construction and Building Materials, 491, 142459. https://doi.org/10.1016/j.conbuildmat.2025.142459

Gursel, A. (2012). Concrete LCA Webtool. https://greenconcrete.berkeley.edu/concretewebtool.html

Gursel, A. P. (2014). Life-Cycle Assessment of Concrete: Decision-Support Tool and Case Study Application [UC Berkeley]. https://escholarship.org/uc/item/5q24d64s

Heidelberg Materials. (2024). Next-level concrete recycling. https://www.heidelbergmaterials.com/en/sustainability/we-decarbonize-the-construction-industry/taking-concrete-recycling-to-the-next-level

Hertel, T., Van den Bulck, A., Blanpain, B., & Pontikes, Y. (2022). Correlating the amorphous phase structure of vitrified bauxite residue (red mud) to the initial reactivity in binder systems. Cement and Concrete Composites, 127, 104410. https://doi.org/10.1016/j.cemconcomp.2022.104410

IPCC. (2000, May 1). Good Practice Guidance and Uncertainty Management in National Greenhouse Gas Inventories. https://www.ipcc.ch/publication/good-practice-guidance-and-uncertainty-management-in-national-greenhouse-gas-inventories/

Kellenberger, D., Althaus, H.-J., Jungbluth, N., Künniger, T., Lehmann, M., & Thalmann, P. (2007). Life cycle inventories of building products. Final Report Ecoinvent Data v2. 0 No, 7. https://www.academia.edu/download/59905670/07_BuildingProducts20190701-59827-1ctcl96.pdf

Koufany, I., De la Torre, A. G., Redondo-Soto, C., Santacruz, I., Cuesta, A., Gastaldi, D., Canonico, F., Mazanec, O., & Aranda, M. A. G. (2025). General correlation between R3 test results and compressive strengths for five families of supplementary cementitious materials. Cement and Concrete Composites, 163, 106166. https://doi.org/10.1016/j.cemconcomp.2025.106166

Li, X., Snellings, R., Antoni, M., Alderete, N. M., Ben Haha, M., Bishnoi, S., Cizer, Ö., Cyr, M., De Weerdt, K., Dhandapani, Y., Duchesne, J., Haufe, J., Hooton, D., Juenger, M., Kamali-Bernard, S., Kramar, S., Marroccoli, M., Joseph, A. M., Parashar, A., … Scrivener, K. L. (2018). Reactivity tests for supplementary cementitious materials: RILEM TC 267-TRM phase 1. Materials and Structures, 51(6), 151. https://doi.org/10.1617/s11527-018-1269-x

Londono-Zuluaga, D., Gholizadeh-Vayghan, A., Winnefeld, F., Avet, F., Ben Haha, M., Bernal, S. A., Cizer, Ö., Cyr, M., Dolenec, S., Durdzinski, P., Haufe, J., Hooton, D., Kamali-Bernard, S., Li, X., Marsh, A. T. M., Marroccoli, M., Mrak, M., Muy, Y., Patapy, C., … Scrivener, K. L. (2022). Report of RILEM TC 267-TRM phase 3: Validation of the R3 reactivity test across a wide range of materials. Materials and Structures, 55(5), 142. https://doi.org/10.1617/s11527-022-01947-3

Marceau, M., & VanGeem, M. G. (2003). Life Cycle Inventory of Slag Manufacturing Process (No. 312012). CTL Inc. Unpublished report, provided by CTL Group with permission from Slag Cement Association.

Ramanathan, S., Perumal, P., Illikainen, M., & Suraneni, P. (2021). Mechanically activated mine tailings for use as supplementary cementitious materials. RILEM Technical Letters, 6, 61–69. https://doi.org/10.21809/rilemtechlett.2021.143

Scrivener, K., Martirena, F., Bishnoi, S., & Maity, S. (2018). Calcined clay limestone cements (LC3). Cement and Concrete Research, Report of UNEP SBCI Working Group on Low-CO_2_ Eco-Efficient Cement-Based Materials, 114, 49–56. https://doi.org/10.1016/j.cemconres.2017.08.017

Snellings, R., & Scrivener, K. L. (2016). Rapid screening tests for supplementary cementitious materials: Past and future. Materials and Structures, 49(8), 3265–3279. https://doi.org/10.1617/s11527-015-0718-z

Sousa, L. N., Zepper, J. C. O., Schollbach, K., & Brouwers, H. J. H. (2024). Improving the reactivity of industrial recycled concrete fines: Exploring mechanical and hydrothermal activation. Construction and Building Materials, 442, 137594. https://doi.org/10.1016/j.conbuildmat.2024.137594

Suraneni, P., Hajibabaee, A., Ramanathan, S., Wang, Y., & Weiss, J. (2019). New insights from reactivity testing of supplementary cementitious materials. Cement and Concrete Composites, 103, 331–338. https://doi.org/10.1016/j.cemconcomp.2019.05.017

Surchiste. (2021, April 1). Our industrial tools. SURSCHISTE. https://surschiste.com/en/industrial-tools/

Wang, Y., & Suraneni, P. (2019). Experimental methods to determine the feasibility of steel slags as supplementary cementitious materials. Construction and Building Materials, 204, 458–467. https://doi.org/10.1016/j.conbuildmat.2019.01.196

Weidema, B. P., & Wesnæs, M. S. (1996). Data quality management for life cycle inventories—An example of using data quality indicators. Journal of Cleaner Production, 4(3), 167–174. https://doi.org/10.1016/S0959-6526(96)00043-1

Yao, Z., Kashani, A., Rawal, A., Song, H., & Kim, T. (2024). Heat-induced phase transitions in mining tailings to create alternative supplementary cementitious materials. Resources, Conservation and Recycling, 210, 107818. https://doi.org/10.1016/j.resconrec.2024.107818
